# On the Evaluation of Rhamnolipid Biosurfactant Adsorption Performance on Amberlite XAD-2 Using Machine Learning Techniques

**DOI:** 10.1155/2021/5530093

**Published:** 2021-04-26

**Authors:** Fengqin Chen, Jinbo Huang, Xianjun Wu, Xiaoli Wu, Arash Arabmarkadeh

**Affiliations:** ^1^Inspection Department, Maoming People's Hospital, Maoming Guangdong 525000, China; ^2^Logistics Department, Maoming People's Hospital, Maoming Guangdong 525000, China; ^3^School of Computer, Guangdong University of Petrochemical Technology, Maoming Guangdong 525000, China; ^4^Burn Department of Maoming People's Hospital, Maoming Guangdong 525000, China; ^5^Biotechnology Group, Faculty of Chemical Engineering, Tarbiat Modares University, P.O. Box 14115-143, Tehran, Iran; ^6^Microbial Biotechnology Department, Agricultural Biotechnology Research Institute of Iran (ABRII), Agricultural Research Education and Extension Organization (AREEO), Karaj, Iran

## Abstract

Biosurfactants are a series of organic compounds that are composed of two parts, hydrophobic and hydrophilic, and since they have properties such as less toxicity and biodegradation, they are widely used in the food industry. Important applications include healthy products, oil recycling, and biological refining. In this research, to calculate the curves of rhamnolipid adsorption compared to Amberlite XAD-2, the least-squares vector machine algorithm has been used. Then, the obtained model is formed by 204 adsorption data points. Various graphical and statistical approaches are applied to ensure the correctness of the model output. The findings of this study are compared with studies that have used artificial neural network (ANN) and data group management method (GMDH) models. The model used in this study has a lower percentage of absolute mean deviation than ANN and GMDH models, which is estimated to be 1.71%.The least-squares support vector machine (LSSVM) is very valuable for investigating the breakthrough curve of rhamnolipid, and it can also be used to help chemists working on biosurfactants. Moreover, our graphical interface program can assist everyone to determine easily the curves of rhamnolipid adsorption on Amberlite XAD-2.

## 1. Introduction

As mentioned above, biosurfactants are organic compounds that are produced by microorganisms and consist of two parts: hydrophilic and hydrophobic. They are often produced by bacteria on living surfaces. One of the reasons for attracting many industrial applications to biosurfactants is due to their amphiphilic properties. Among the usable and outstanding capabilities of biosurfactants used in various industries such as mines, fertilizers, petrochemicals, and petroleum, we can mention the environmental degradability and reduction of surface tension between interstitial and low toxicity. The reduction of the interfacial tension is due to the increase in the solubility of hydrophilic molecules when using biosurfactants. Capabilities such as surface modification and interfacial tension have made surfactants attractive to the industry. Rhamnolipids (RLs) are the most studied type of biosurfactants. According to the literature, rhamnolipids can reduce water surface tension by about 60% [1–3] for different concentrations of RL 50-65 mg/L. The production of RLs usually involves a final product from a dilute solution contaminated with undesirable impurities. There are several ways to increase the concentration and eliminate contaminants in which the adsorption process is widely studied.

In this research, activated carbon is used for adsorbent in the process. The breakthrough curve of a packed column is a very significant attribute of this system. As a result, determining such curves will be useful for optimizing and understanding the performance of the column. To model the adsorption phenomena, the mass balance in liquid and solid phases is evaluated. It may also include modeling the porous and liquid film resistance and also axial dispersion. Finally, with a suitable software package, a set of differential equations can be solved.

Ill conditions and uncertainty in differential equations make using conventional mathematical models not suitable. Intelligent models have to be a powerful tool in solving process modeling problems. To predict the optimized targets, in various fields such as petroleum and gas fields, methods such as SVM, ANN, group method data manipulation (GMDH), fuzzy logic system, and adaptive fuzzy neural inference system can be used. Interactions between AI neurons are achieved by connecting different units. Artificial neurons' interactions are achieved by connecting different units. Each weighted output is related to the sum of the output from the previous synaptic weight layer, and then it is used as an input for a specific neuron. Backpropagation ANNs are extensively applied, as they have shown to be a capable and powerful tool [4]. The GMDH model is a type of backpropagation ANN that was proposed by Ivakhnenko [5]. Darwin's theory of selection inspired this approach. The prominent feature of this method is the internal process of the elements [6–8]. To process elements in a conventional ANN, log sigmoid, hard limit, linear, and tangent sigmoid transfer functions are considered. On the other hand, the GMDH method constructs simple polynomials, roughly predicting the targeted systems. In the next step, the complexity of the polynomials is further developed so that satisfactory models are achieved. [9, 10].

**Figure 1:**
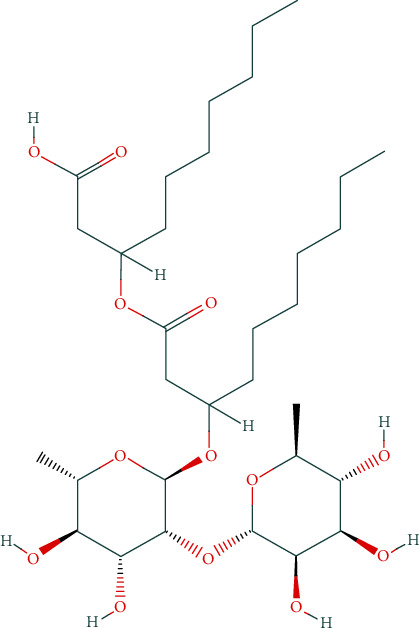
Chemical structure of the first identified rhamnolipid, symbolized as Rha-Rha-C_10_-C_10_.

Due to the importance of predicting a trustworthy estimation of breakthrough curves, this research is aimed at predicting of breakthrough curves utilizing the LSSVM method for rhamnolipid (Figure 1) adsorption over Amberlite XAD-2. Furthermore, results are compared with those of ANN and GMDH models. The investigated model takes into account 204 data points in its network for adsorption over the Amberlite XAD-2. Various graphical and statistical methods are considered to evaluate the accuracy of this strategy.

## 2. Model Development

In the present research, the LSSVM strategy was applied to calculate the curve to achieve rhamnolipid uptake relative to the Amberlite XAD-2 model resulting in a more simplified way [11, 12]. SVM can be defined as a function as below:

(1) $$\begin{array}{c} f\left(x\right)=w^{T}\varphi \left(x\right)+b. \end{array}$$

The parameters of the above expression are as follows:

*w* ^*T*^ denotes the transpose vector corresponding tothe output layer.

*b* and *φ*(*x*) represent the bias and the kernel function, respectively.

The input (*x*) consists of *N* × *n* dimension in which *n* and N are input parameters and some data points, respectively. The following cost function is optimized to evaluate *w*^*T*^ and *b* parameters [13]:

(2) $$\begin{array}{c} \text{cost function}=\frac{1}{2}w^{T}+C\overset{N}{\underset{k=1}{\sum }}\left(\xi_{k}-\xi_{k}^{*}\right), \end{array}$$

which is constrained by

(3) $$\begin{array}{c} \left\{\begin{array}{c} {\text{y}}_{\text{k}}-{\text{w}}^{\text{T}}\varphi \left({\text{x}}_{\text{k}}\right)-\text{b}\leq \varepsilon +\xi_{\text{k}},\text{k}=1,2,\cdots ,\text{N}\\ {\text{w}}^{\text{T}}\varphi \left({\text{x}}_{\text{k}}\right)+\text{b}-{\text{y}}_{\text{k}}\leq \varepsilon +\xi_{\text{k}}^{*},\text{k}=1,2,\cdots ,\text{N}\\ \xi_{\text{k}},\xi_{\text{k}}^{*}\geq 0 \end{array}\right. \end{array}$$

*y*_*k*_ is the *k*^th^ output while *x*_*k*_ is the *k*^th^ input. *ε* stands for the fixed precision of the estimation. Also, slack variables (*ξ*_*k*_, *ξ*_*k*_^∗^) are dealing to determine the acceptable error margin. The below lagrangian is applied to minimize the cost function:

(4) $$\begin{array}{c} L\left(a,a^{*}\right)=-\frac{1}{2}\overset{N}{\underset{k,l=1}{\sum }}\left(a_{k}-a_{k}^{*}\right)\left(a_{l}-a_{l}^{*}\right)K\left(x_{k},x_{l}\right)-\varepsilon \overset{N}{\underset{k=1}{\sum }}\left(a_{k}-a_{k}^{*}\right)+\overset{N}{\underset{k=1}{\sum }}y_{k}\left(a_{k}-a_{k}^{*}\right),\\ \overset{N}{\underset{k=1}{\sum }}\left(a_{k}-a_{k}^{*}\right)=0,a_{k},a_{k}^{*}\in \left[0,c\right],\\ K\left(x_{k},x_{l}\right)=\varphi {\left(x_{k}\right)}^{T}\varphi \left(x_{l}\right),k=1,2,\cdots ,N, \end{array}$$

where *a*_*k*_ and *a*_*k*_^∗^ stand for Lagrangian multipliers. In the last step, the SVM is given below:

(5) $$\begin{array}{c} f\left(x\right)=\overset{N}{\underset{k,l=1}{\sum }}\left(a_{k}-a_{k}^{*}\right)K\left(x,x_{k}\right)+b. \end{array}$$

Quadratic programming must be solved to determine the SVM parameters. The LSSVM eliminates deficiencies in the solving process of a quadratic programming problem [11, 12]. LSSVM uses the below equation in the process of model development:

(6) $$\begin{array}{c} \text{cost function}=\frac{1}{2}w^{T}w+\frac{1}{2}\gamma \overset{N}{\underset{k=1}{\sum }}e_{k}^{2}, \end{array}$$

where

*γ* denotes tuning parameter.

*e*_*k*_ is the error variable.

The following constraints are applied to the cost function:

(7) $$\begin{array}{c} y_{k}=w^{T}\varphi \left(x_{k}\right)+b+e_{k}. \end{array}$$

The Lagrangian of the LSSVM is expressed as

(8) $$\begin{array}{c} l\left(w,b,e,a\right)=\frac{1}{2}w^{T}w+\frac{1}{2}\gamma \overset{N}{\underset{k=1}{\sum }}e_{k}^{2}-\overset{N}{\underset{k=1}{\sum }}a_{k}\left(w^{T}\varphi \left(x_{k}\right)+b+e_{k}-y_{k}\right). \end{array}$$

In the above phrase, the symbol *a*_*k*_ represents the Lagrangian multipliers. To optimize Eq. (8), its derivatives are set to zero, and as a result, the following equations are achieved:

(9) $$\begin{array}{c} \left\{\begin{array}{c} \frac{\partial L}{\partial w}=0⟹w=\overset{N}{\underset{k=1}{\sum }}a_{k}\varphi \left(x_{k}\right),\\ \frac{\partial L}{\partial b}=0⟹\overset{N}{\underset{k=1}{\sum }}a_{k}=0,\\ \frac{\partial L}{\partial e_{k}}=0⟹a_{k}=\gamma e_{k},k=1,2,\cdots ,N,\\ \frac{\partial L}{\partial a_{k}}=0⟹w^{T}\varphi \left(x_{k}\right)+b+e_{k}-y_{k}=0,k=1,2,\cdots ,N. \end{array}\right. \end{array}$$

**Figure 2:**
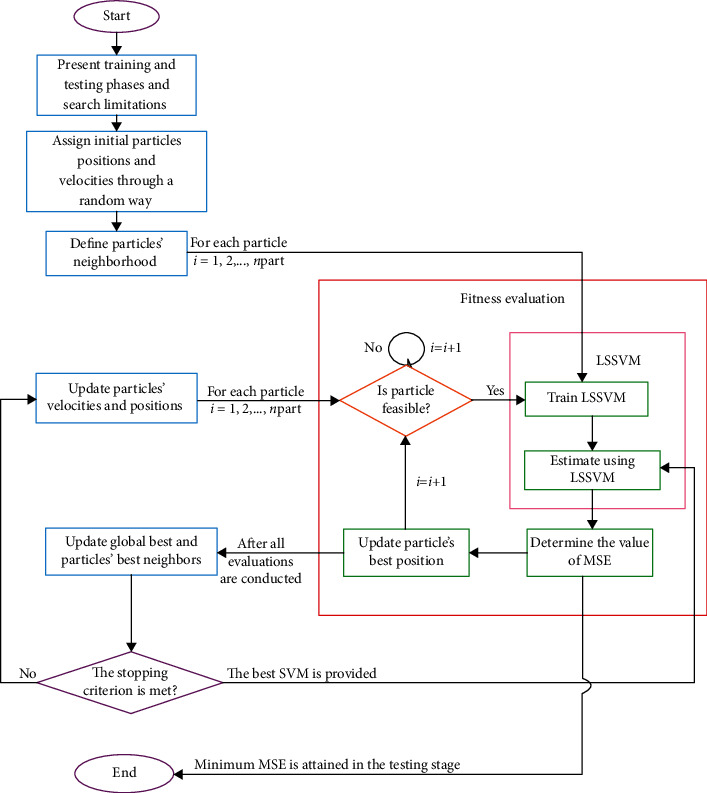
Schematic diagram of PSO-LSSVM strategy.

By solving the aforementioned equations, LSSVM parameters are obtained. LSSVM employs the kernel function in the same way that SVM strategy does. The most common applied kernel function is the radial basis function (RBF) which is given by

(10) $$\begin{array}{c} K\left(x,x_{k}\right)=\exp\left(-{\left\|x_{k}-x\right\|}^{2}/\sigma^{2}\right), \end{array}$$

where *σ*^2^ stands for the tuning parameter corresponding to the kernel function. As a result, two tuning parameters (*σ*^2^ and *γ*) are adjustable. The last-mentioned parameters can be determined by minimizing the error between the predicted values and experimental ones through the application of mean square error (MSE):

(11) $$\begin{array}{c} MSE=\frac{1}{N}\overset{N}{\underset{k=1}{\sum }}{\left(y_{k}^{\text{pred}.}-y_{k}^{\exp.}\right)}^{2}, \end{array}$$

where *y* is the output value, and exp. and pred. subscripts denote experimental and predicted values, respectively. Also, in this paper, we used the particle swarm optimization algorithm for the determination of these tuning parameters. A typical diagram of the proposed LSSVM approach has been shown in Figure 2.

The adjusted parameters are *γ* and *σ*^2^ in the LSSVM model and based on the identified cost function (Eq. (11)), and these parameters are optimally determined by optimization technique. The values of *γ* and *σ*^2^ in this study are 984523.52 and 0.246, respectively, through the PSO algorithm with swarm size and iteration of 80 and 1000, respectively.

Different statistical error analyses such as mean absolute error (MAE), coefficient of determination (*R*^2^), and root means square error (RMSE) are implicated to analyze the model's performance.

(12) $$\begin{array}{c} R^{2}=1-\frac{\sum_{k=1}^{N}{\left(y_{k}^{\exp.}-y_{k}^{\text{pred}.}\right)}^{2}}{\sum_{k=1}^{N}{\left(y_{k}^{\exp.}-y_{\text{ave}.}\right)}^{2}},\\ \text{MAE}=\frac{\sum_{k=1}^{N}\left|y_{k}^{\text{pred}.}-y_{k}^{\exp.}\right|}{N},\\ \text{RMSE}=\sqrt{\frac{1}{N}\overset{N}{\underset{k=1}{\sum }}{\left(y_{k}^{\text{pred}.}-y_{k}^{\exp.}\right)}^{2}}. \end{array}$$

## 3. Identification of Outlying Experimental Data

The outlier is a set of data having a different behavior in comparison with the bulk of data. Finding outliers would improve the accuracy and reliability of a proposed model remarkably. To help to trace outliers, there are two procedures numerical and graphical procedures. One of the most powerful methods is the Leverage method in which the deviation of estimated values from the experimental ones is calculated. It also includes dealing with Hat matrix being made of experimental and predicted data. The equation below is used for calculating the Hat indices [14–16]:

(13) $$\begin{array}{c} H=X{\left(X^{t}X\right)}^{-1}X^{t}. \end{array}$$

*X* (*n* × *x*) is a matrix including *n* data and *k* parameters of the model, and *t* denotes the transpose matrix. The diagonal values of the matrix (*H*) are called *H* values. *H* values will aid in the detection process of the possible outliers, utilizing a Williams plot in which the relationship between standardized crossvalidated residuals (*R*) and Hat indices is shown. The warning leverage is given as follows:

(14) $$\begin{array}{c} H^{*}=3\frac{\left(f+1\right)}{p}. \end{array}$$

*p* and *f* stand for numbers of data points and model parameters, respectively.

A reliable model would contain the majority of the predicted values by satisfying the following constraint:

(15) $$\begin{array}{c} R\in \left[-3,3\right],0<H<{\text{H}}^{*}. \end{array}$$

Regardless of the value of *H*, if the value of *R* for a given data is outside the above range, it is considered a possible candidate for being an outlier. The data in this paper are provided in Table S1, and this data set is taken from the previous paper [17]. As discussed, input parameters are initial rhamnolipid concentration, fixed bed height, flow velocity, and run time, while the ratio of final to initial concentration of rhamnolipid (C/C_0) would be the output parameters ones. The 204 data points are divided into two categories: training and testing.

To create the LSSVM model, 75% of data points are considered as learning points, and the rest of them were used to examine the efficiency of the opposed model. Furthermore, data are normalized within the range of [-1,1] applying the equation below:

(16) $$\begin{array}{c} D_{N}=2\frac{D-D_{\min}}{D_{\max}-D_{\min}}-1. \end{array}$$

Here, *D* and *D*_*N*_ represent actual and normalized data points, respectively. Also, *D*_min_ and *D*_max_ stand for minimum and maximum values of data points, respectively.

### 3.1. Evaluation of the model's Accuracy

**Figure 3:**
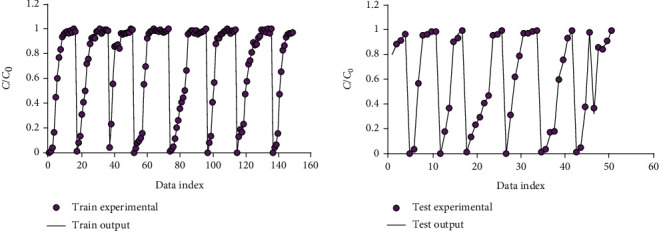
Plot of PSO-LSSVM model's prediction vs. experimental data at training and testing stages.

The predictive model's accuracy is investigated employing different graphical and statistical methods.  Figure 3 represents experimental data points and model estimation by the proposed LSSVM method in the training and testing stages.

**Figure 4:**
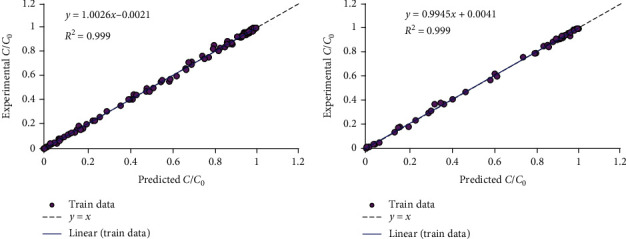
Regression plot of suggested PSO-LSSVM model at training and testing stages.

Figure 4 shows predicted values against experimental ones. The more it would be close to line *Y* = *X*, the more appropriate the prediction of the proposed model.

Results of the current study are compared to the LSSVM, ANN, and GMDH models [17]. To estimate the curvature of rhamnolipids on activated carbon, the structures of the proposed GMDH model are given below:

(i) First layer:
  (17) $$\begin{array}{c} \text{Node }1:z_{1}=0.183x_{1}^{2}+0.149x_{2}^{2}+0.165x_{1}x_{2}+-0.242x_{1}-0.048x_{2}+0.678\\ \begin{array}{cc} \text{Node }2: & z_{2}=0.141x_{2}^{2}-1.6x_{4}^{2}-0.21x_{2}x_{4}-0.028x_{2}+2.76x_{4}-0.167 \end{array}\\ \begin{array}{cc} \text{Node }3: & z_{3}=0.182x_{3}^{2}-1.606x_{4}^{2}-0.124x_{3}x_{4}-0.075x_{3}+2.778x_{4}-0.167 \end{array}\\ \begin{array}{cc} \text{Node }4: & z_{4}=-0.002x_{1}^{2}-1.64x_{4}^{2}+0.172x_{1}x_{4}-0.169x_{1}2.684x_{4}-0.075 \end{array} \end{array}$$
(ii) Second layer:
  (18) $$\begin{array}{c} \begin{array}{cc} \text{Node }1: & w_{1}=-0.253z_{1}^{2}+0.248z_{2}^{2}-0.319z_{1}z_{2}-0.05z_{1}+0.948z_{2}-0.044 \end{array}\\ \begin{array}{cc} \text{Node }2: & w_{2}=4.923z_{1}^{2}+0.234z_{3}^{2}-0.057z_{1}z_{3}-5.738z_{1}+0.794z_{3}+1.676 \end{array}\\ \begin{array}{cc} \text{Node }3: & w_{3}=2.915z_{1}^{2}+0.237z_{4}^{2}-0.535z_{1}z_{4}-2.733z_{1}+1.097z_{4}+0.577 \end{array} \end{array}$$
(iii) Third layer:
  (19) $$\begin{array}{c} \begin{array}{cc} \text{Node }1: & u_{1}=-0.802w_{1}^{2}-1.248w_{2}^{2}+1.972w_{1}w_{2}+0.419w_{1}+0.666w_{2}-0.011 \end{array}\\ \begin{array}{cc} \text{Node }2: & u_{2}=1.433w_{2}^{2}+1.571w_{3}^{2}-3.077w_{2}w_{3}+0.603w_{2}+0.48w_{3}-0.014 \end{array} \end{array}$$
(iv) Genome expression:
  (20) $$\begin{array}{c} \begin{array}{cc} \text{Node }1: & \frac{C}{C_{0}}=-0.802u_{1}^{2}-1.24u_{2}^{2}-1.972u_{1}u_{2}+0.419u_{1}+0.666u_{2}-0.011 \end{array} \end{array}$$

The ANN model based on these four input variables as mentioned as follows:

1. input layer
2. hidden layer including six neurons
3. output layer

**Figure 5:**
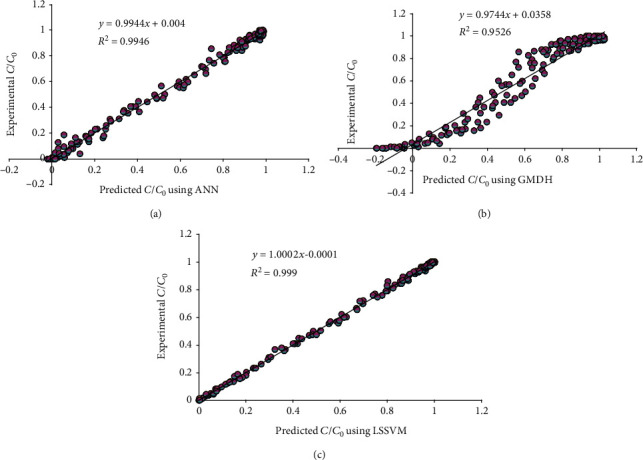
Cross plot of predictions of different models for total data points: (a) ANN, (b) GMDH, and (c) LSSVM.

Figure 5 represents the cross plot of the aforementioned strategies. As explained, data points of the LSSVM model are closer to the line *Y* = *X*, than ANN and GMDH models. Also, the calculation of the determination coefficient shows that the proposed LSSVM approach is superior to ANN and GMDH in terms of accuracy.

**Figure 6:**
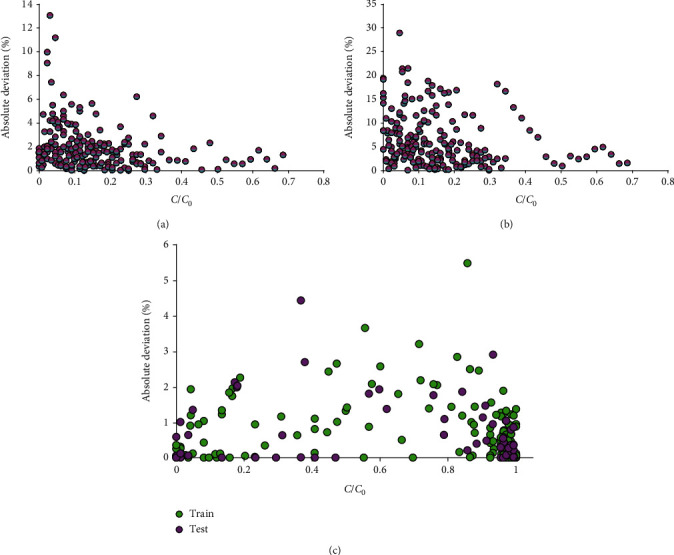
Absolute error for different models' outcomes: (a) ANN, (b) GMDH, and (c) LSSVM.

Compared to ANN and GMDH models, the less relative error is observed in the proposed LSSVM model.  Figure 6 indicates more reliability of the suggested LSSVM model.

Estimation accuracy is also investigated by applying the following statistical methods:

(21) $$\begin{array}{c} R^{2}=1-\frac{\sum_{i=1}^{N}{\left(C_{\text{Pred}}\left(i\right)-C_{\text{Exp}}\left(i\right)\right)}^{2}}{\sum_{i=1}^{N}{\left(C_{\text{Pred}}\left(i\right)-{\bar{C}}_{\text{Exp}}\left(i\right)\right)}^{2}},\\ \%\text{AAD}=\frac{100}{N}\overset{N}{\underset{i=1}{\sum }}C_{\text{Pred}}\left(i\right)-C_{\text{Exp}}\left(i\right),\\ \text{MSE}=\left(\frac{\sum_{i=1}^{N}{\left(C_{\text{Pred}}\left(i\right)-C_{\text{Exp}}\left(i\right)\right)}^{2}}{N}\right),\\ \text{STD}=\overset{n}{\underset{i=1}{\sum }}{\left(\frac{{\left(C_{\text{Pred}}\left(i\right)-{\bar{C}}_{\text{Exp}}\left(i\right)\right)}^{2}}{N}\right)}^{0.5}. \end{array}$$

**Figure 7:**
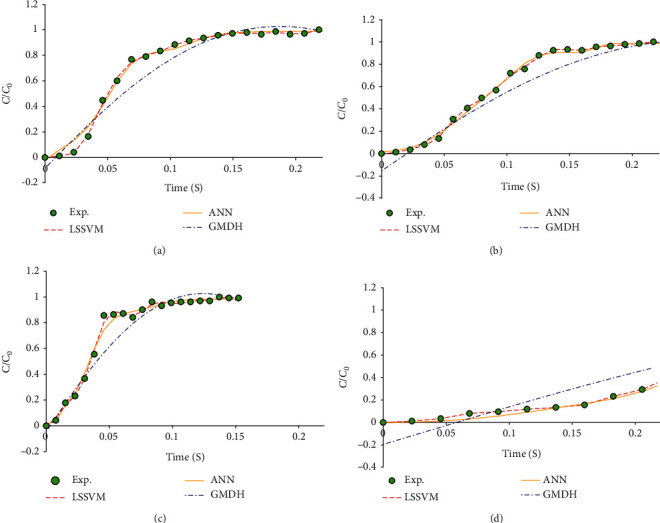
Predicted *C*/*C*_0_ as a function of time by the three models for (a) *H*_0_ = 7, *U* = 160, and *C*_0_ = 24, (b) *H*_0_ = 11, *U* = 160, and *C*_0_ = 8, (c) *H*_0_ = 7, *U* = 240, and *C*_0_ = 24, and (d) *H*_0_ = 11, *U* = 80, and *C*_0_ = 8.

Table 1 presents statistical values of the presented model compared with ANN and GMDH approaches showing the higher value of *R*^2^ and lower values of STD, AAD, and RMSE, and as a result, the LSSVM model possesses higher accuracy and reliability than others. The dependency of the (*C*/*C*_0_) as an output parameter on input parameters is illustrated in Figure 7.

Four different conditions of *H*_0_ = 7, *U* = 160, and *C*_0_ = 24, and *H*_0_ = 11, *U* = 160, and *C*_0_ = 8 and *H*_0_ = 7, *U* = 240, and *C*_0_ = 24 and *H*_0_ = 11, *U* = 80, and *C*_0_ = 8 were investigated to measure the prediction ability of LSSVM, ANN, and GMDH models for indicating that the LSSVM model acquired better estimation. As this figure shows, as time goes by, the ratio of *C*/*C*_0_ increases.

**Figure 8:**
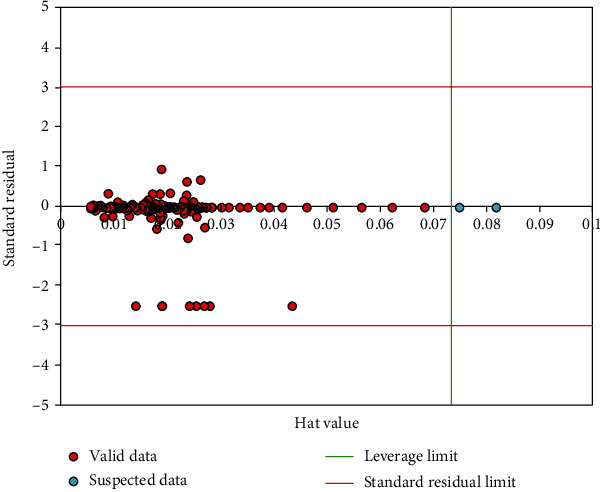
Diagnosis of the probable outlier data and applicability domain of the applied model.

In the last part of this research, the leverage approach is applied to find outliers, employing the Hat matrix, Williams plot, and residuals. As discussed, Eq. (13) is used to calculate *H* values.  Figure 8 also illustrates the Williams plot. All of the *H* is in the range [-3, +3], and *R* is in the range [0, 0.08], and then the accuracy of the proposed model is desirable and acceptable; so, the accuracy of the proposed model is satisfactory. There are only two of the data points that are outside of the applicable domain which is shown in the figure by a blue circle. As *R* values approach zero and *H* value reduces, the reliability of data points is increased [18–23].

## 4. Conclusion

Then LSSVM approach was employed to estimate breakthrough curves of rhamnolipid adsorption over Amberlite XAD-2 as a function of fixed bed height, flow velocity, runtime, and initial rhamnolipid concentration. The particle swarm optimization method was employed for the training process enhancing the accuracy of the proposed model. Various statistical and graphical methods were applied to evaluate the model's reliability showing that the AAD% value for adsorption over activated carbon was 0.75%. For ANN and GMDH models that were developed by Padilha et al., AAD% of activated carbon is reported to be 1.9% and 6.2%. Based on the above evidence, we can find that the proposed LSSVM model is more reliable for the process of predicting the breakthrough curves.

## Figures and Tables

**Figure 9:**
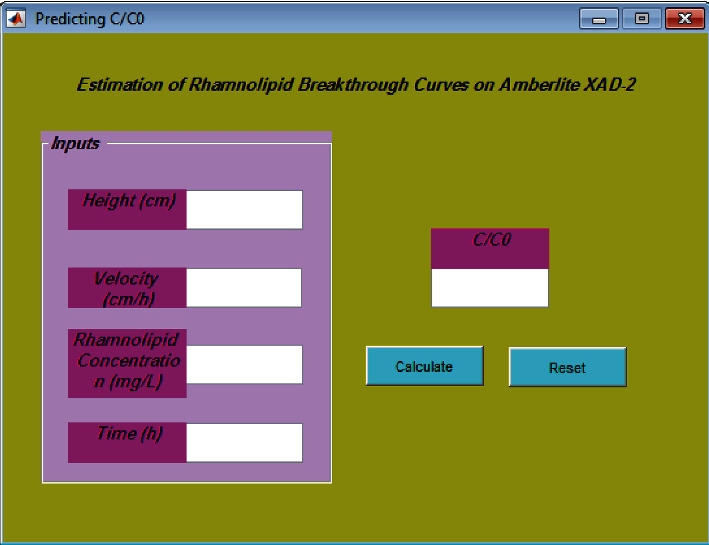
GUI version of the developed LSSVM model.

**Table 1:** Statistical parameters calculated for three models.

Analysis	LSSVM			ANN	GMDH
	Train	Test	Total	Total	Total
MSE	0.0001	0.0002	0.0001	0.0005	0.0047
AAD	0.7293	0.8043	0.7481	1.9111	6.2395
*R* ^2^	0.9990	0.9990	0.9990	0.9946	0.9526
STD	0.0112	0.0122	0.0115	0.0271	0.0808

## Data Availability

The datasets generated during and/or analyzed during the current study are available from the corresponding author on reasonable request.

## Appendix

### Instructions of the Developed Program

A graphical user interface (GUI) version of the model is developed (Figure 9). The code is compiled to an Exe file which is given in the supplementary content. Matlab software must be installed before running the code. It starts by giving four parameters as input; then, to show the output result, it is enough to click on the calculate button.

## Supplementary Materials

Supplementary materials Table S1: experimental data points used in this study.
