# Supplementary material for: On the Evaluation of Rhamnolipid Biosurfactant Adsorption Performance on Amberlite XAD-2 Using Machine Learning Techniques
Source: Biomed Res Int. 2021 Apr 26;2021:5530093. doi: 10.1155/2021/5530093 (PMC8096566; doi:10.1155/2021/5530093)
Supplement: Supplementary materials — Table S1: experimental data points used in this study. [file 5530093.f1.docx]

**Supplementary Data:**

**Table S1:** Experimental data points used in this study

| ***H_0_* (cm)** | ***u*(cm/h)** | ***C_0_*(mg/L)** | ***t* (h)** | ***C/C_0_*** | ***H_0_* (cm)** | ***u*(cm/h)** | ***C_0_*(mg/L)** | ***t* (h)** | ***C/C_0_*** |
| --- | --- | --- | --- | --- | --- | --- | --- | --- | --- |
| 7 | 160 | 24 | 0 | 0 | 11 | 160 | 16 | 0.022836 | 0.02672 |
| 7 | 160 | 24 | 0.011505 | 0.011996 | 11 | 160 | 16 | 0.034254 | 0.048673 |
| 7 | 160 | 24 | 0.02301 | 0.041055 | 11 | 160 | 16 | 0.045673 | 0.114534 |
| 7 | 160 | 24 | 0.034515 | 0.164555 | 11 | 160 | 16 | 0.057091 | 0.202349 |
| 7 | 160 | 24 | 0.046021 | 0.44788 | 11 | 160 | 16 | 0.068509 | 0.260892 |
| 7 | 160 | 24 | 0.057526 | 0.60044 | 11 | 160 | 16 | 0.079927 | 0.312118 |
| 7 | 160 | 24 | 0.069031 | 0.767528 | 11 | 160 | 16 | 0.091345 | 0.356025 |
| 7 | 160 | 24 | 0.080536 | 0.789323 | 11 | 160 | 16 | 0.102763 | 0.40725 |
| 7 | 160 | 24 | 0.092041 | 0.832911 | 11 | 160 | 16 | 0.114181 | 0.44384 |
| 7 | 160 | 24 | 0.103546 | 0.883764 | 11 | 160 | 16 | 0.125599 | 0.502383 |
| 7 | 160 | 24 | 0.115051 | 0.912823 | 11 | 160 | 16 | 0.137018 | 0.619469 |
| 7 | 160 | 24 | 0.126556 | 0.934617 | 11 | 160 | 16 | 0.148436 | 0.663377 |
| 7 | 160 | 24 | 0.138062 | 0.956412 | 11 | 160 | 16 | 0.159854 | 0.787781 |
| 7 | 160 | 24 | 0.149567 | 0.970941 | 11 | 160 | 16 | 0.171272 | 0.956093 |
| 7 | 160 | 24 | 0.161072 | 0.978206 | 11 | 160 | 16 | 0.18269 | 0.970728 |
| 7 | 160 | 24 | 0.172577 | 0.963676 | 11 | 160 | 16 | 0.194108 | 0.970728 |
| 7 | 160 | 24 | 0.184082 | 0.985471 | 11 | 160 | 16 | 0.205526 | 0.985364 |
| 7 | 160 | 24 | 0.195587 | 0.963676 | 11 | 160 | 16 | 0.216945 | 0.985364 |
| 7 | 160 | 24 | 0.207092 | 0.970941 | 11 | 160 | 16 | 0.228363 | 0.963411 |
| 7 | 160 | 24 | 0.218597 | 1 | 11 | 160 | 16 | 0.239781 | 0.985364 |
| 7 | 160 | 24 | 0.230103 | 0.978206 | 11 | 160 | 16 | 0.251199 | 0.956093 |
| 11 | 160 | 8 | 0 | 0 | 11 | 160 | 16 | 0.262617 | 0.970728 |
| 11 | 160 | 8 | 0.011418 | 0.012543 | 11 | 160 | 16 | 0.274035 | 0.970728 |
| 11 | 160 | 8 | 0.022836 | 0.03533 | 11 | 160 | 16 | 0.285453 | 0.970728 |
| 11 | 160 | 8 | 0.034254 | 0.080905 | 11 | 160 | 16 | 0.296871 | 0.985364 |
| 11 | 160 | 8 | 0.045673 | 0.134076 | 11 | 160 | 16 | 0.30829 | 0.992682 |
| 11 | 160 | 8 | 0.057091 | 0.30878 | 11 | 160 | 16 | 0.319708 | 0.978046 |
| 11 | 160 | 8 | 0.068509 | 0.407526 | 11 | 160 | 16 | 0.331126 | 0.985364 |
| 11 | 160 | 8 | 0.079927 | 0.498676 | 11 | 160 | 16 | 0.342544 | 1 |
| 11 | 160 | 8 | 0.091345 | 0.567038 | 9 | 160 | 8 | 0 | 0 |
| 11 | 160 | 8 | 0.102763 | 0.718954 | 9 | 160 | 8 | 0.011418 | 0.012543 |
| 11 | 160 | 8 | 0.114181 | 0.756934 | 9 | 160 | 8 | 0.022836 | 0.03533 |
| 11 | 160 | 8 | 0.125599 | 0.878467 | 9 | 160 | 8 | 0.034254 | 0.080905 |
| 11 | 160 | 8 | 0.137018 | 0.924042 | 9 | 160 | 8 | 0.045673 | 0.134076 |
| 11 | 160 | 8 | 0.148436 | 0.931638 | 9 | 160 | 8 | 0.057091 | 0.172055 |
| 11 | 160 | 8 | 0.159854 | 0.924042 | 9 | 160 | 8 | 0.068509 | 0.179651 |
| 11 | 160 | 8 | 0.171272 | 0.954425 | 9 | 160 | 8 | 0.079927 | 0.407526 |
| 11 | 160 | 8 | 0.18269 | 0.962021 | 9 | 160 | 8 | 0.091345 | 0.567038 |
| 11 | 160 | 8 | 0.194108 | 0.977213 | 9 | 160 | 8 | 0.102763 | 0.597421 |
| 11 | 160 | 8 | 0.205526 | 0.984808 | 9 | 160 | 8 | 0.114181 | 0.756934 |
| 11 | 160 | 8 | 0.216945 | 1 | 9 | 160 | 8 | 0.125599 | 0.878467 |
| 11 | 160 | 8 | 0.228363 | 0.984808 | 9 | 160 | 8 | 0.137018 | 0.924042 |
| 11 | 160 | 8 | 0.239781 | 0.962021 | 9 | 160 | 8 | 0.148436 | 0.931638 |
| 11 | 160 | 8 | 0.251199 | 0.984808 | 9 | 160 | 8 | 0.159854 | 0.924042 |
| 11 | 160 | 8 | 0.262617 | 0.977213 | 9 | 160 | 8 | 0.171272 | 0.954425 |
| 11 | 160 | 8 | 0.274035 | 0.992404 | 9 | 160 | 8 | 0.18269 | 0.962021 |
| 11 | 160 | 8 | 0.285453 | 0.992404 | 9 | 160 | 8 | 0.194108 | 0.977213 |
| 11 | 160 | 8 | 0.296871 | 0.984808 | 9 | 160 | 8 | 0.205526 | 0.984808 |
| 7 | 240 | 24 | 0 | 0 | 9 | 160 | 8 | 0.216945 | 1 |
| 7 | 240 | 24 | 0.007612 | 0.042602 | 9 | 160 | 8 | 0.228363 | 0.984808 |
| 7 | 240 | 24 | 0.015224 | 0.178297 | 9 | 160 | 8 | 0.239781 | 0.962021 |
| 7 | 240 | 24 | 0.022836 | 0.231066 | 9 | 160 | 8 | 0.251199 | 0.984808 |
| 7 | 240 | 24 | 0.030448 | 0.366761 | 9 | 160 | 8 | 0.262617 | 0.977213 |
| 7 | 240 | 24 | 0.03806 | 0.555225 | 9 | 160 | 8 | 0.274035 | 0.992404 |
| 7 | 240 | 24 | 0.045673 | 0.856767 | 9 | 160 | 8 | 0.285453 | 0.992404 |
| 7 | 240 | 24 | 0.053285 | 0.864306 | 9 | 240 | 8 | 0 | 0 |
| 7 | 240 | 24 | 0.060897 | 0.871844 | 9 | 240 | 8 | 0.007612 | 0.012084 |
| 7 | 240 | 24 | 0.068509 | 0.84169 | 9 | 240 | 8 | 0.015224 | 0.048673 |
| 7 | 240 | 24 | 0.076121 | 0.901999 | 9 | 240 | 8 | 0.022836 | 0.12917 |
| 7 | 240 | 24 | 0.083733 | 0.962307 | 9 | 240 | 8 | 0.030448 | 0.187713 |
| 7 | 240 | 24 | 0.091345 | 0.932153 | 9 | 240 | 8 | 0.03806 | 0.16576 |
| 7 | 240 | 24 | 0.098957 | 0.954769 | 9 | 240 | 8 | 0.045673 | 0.231621 |
| 7 | 240 | 24 | 0.106569 | 0.962307 | 9 | 240 | 8 | 0.053285 | 0.377979 |
| 7 | 240 | 24 | 0.114181 | 0.962307 | 9 | 240 | 8 | 0.060897 | 0.473111 |
| 7 | 240 | 24 | 0.121793 | 0.969846 | 9 | 240 | 8 | 0.068509 | 0.575562 |
| 7 | 240 | 24 | 0.129406 | 0.969846 | 9 | 240 | 8 | 0.076121 | 0.714602 |
| 7 | 240 | 24 | 0.137018 | 1 | 9 | 240 | 8 | 0.083733 | 0.743874 |
| 7 | 240 | 24 | 0.14463 | 0.992461 | 9 | 240 | 8 | 0.091345 | 0.809735 |
| 7 | 240 | 24 | 0.152242 | 0.992461 | 9 | 240 | 8 | 0.098957 | 0.890232 |
| 11 | 80 | 8 | 0 | 0 | 9 | 240 | 8 | 0.106569 | 0.868278 |
| 11 | 80 | 8 | 0.022836 | 0.012543 | 9 | 240 | 8 | 0.114181 | 0.875596 |
| 11 | 80 | 8 | 0.045673 | 0.03533 | 9 | 240 | 8 | 0.121793 | 0.926821 |
| 11 | 80 | 8 | 0.068509 | 0.080905 | 9 | 240 | 8 | 0.129406 | 0.941457 |
| 11 | 80 | 8 | 0.091345 | 0.096097 | 9 | 240 | 8 | 0.137018 | 0.992682 |
| 11 | 80 | 8 | 0.114181 | 0.118884 | 9 | 240 | 8 | 0.14463 | 0.978046 |
| 11 | 80 | 8 | 0.137018 | 0.134076 | 9 | 240 | 8 | 0.152242 | 0.992682 |
| 11 | 80 | 8 | 0.159854 | 0.156863 | 9 | 240 | 8 | 0.159854 | 0.970728 |
| 11 | 80 | 8 | 0.18269 | 0.232822 | 9 | 240 | 8 | 0.167466 | 1 |
| 11 | 80 | 8 | 0.205526 | 0.293588 | 9 | 240 | 8 | 0.175078 | 0.978046 |
| 11 | 80 | 8 | 0.228363 | 0.407526 | 9 | 240 | 8 | 0.18269 | 0.963411 |
| 11 | 80 | 8 | 0.251199 | 0.468292 | 9 | 240 | 8 | 0.190302 | 1 |
| 11 | 80 | 8 | 0.274035 | 0.551846 | 7 | 240 | 8 | 0 | 0 |
| 11 | 80 | 8 | 0.296871 | 0.696167 | 7 | 240 | 8 | 0.007612 | 0.042602 |
| 11 | 80 | 8 | 0.319708 | 0.924042 | 7 | 240 | 8 | 0.015224 | 0.065218 |
| 11 | 80 | 8 | 0.342544 | 0.954425 | 7 | 240 | 8 | 0.022836 | 0.155681 |
| 11 | 80 | 8 | 0.36538 | 0.962021 | 7 | 240 | 8 | 0.030448 | 0.366761 |
| 11 | 80 | 8 | 0.388217 | 0.977213 | 7 | 240 | 8 | 0.03806 | 0.472301 |
| 11 | 80 | 8 | 0.411053 | 0.984808 | 7 | 240 | 8 | 0.045673 | 0.653226 |
| 11 | 80 | 8 | 0.433889 | 1 | 7 | 240 | 8 | 0.053285 | 0.826613 |
| 11 | 80 | 8 | 0.456725 | 0.984808 | 7 | 240 | 8 | 0.060897 | 0.856767 |
| 11 | 80 | 8 | 0.479562 | 0.962021 | 7 | 240 | 8 | 0.068509 | 0.84169 |
| 11 | 80 | 8 | 0.502398 | 0.984808 | 7 | 240 | 8 | 0.076121 | 0.864306 |
| 11 | 80 | 8 | 0.525234 | 0.977213 | 7 | 240 | 8 | 0.083733 | 0.909537 |
| 11 | 80 | 8 | 0.54807 | 0.992404 | 7 | 240 | 8 | 0.091345 | 0.932153 |
| 11 | 80 | 8 | 0.570907 | 0.992404 | 7 | 240 | 8 | 0.098957 | 0.954769 |
| 11 | 80 | 8 | 0.593743 | 0.977213 | 7 | 240 | 8 | 0.106569 | 0.962307 |
| 11 | 80 | 8 | 0.616579 | 0.969617 | 7 | 240 | 8 | 0.114181 | 0.962307 |
| 11 | 80 | 8 | 0.639415 | 0.977213 | 7 | 240 | 8 | 0.121793 | 0.969846 |
| 11 | 80 | 8 | 0.662252 | 0.984808 | 7 | 240 | 8 | 0.129406 | 0.969846 |
| 11 | 80 | 8 | 0.685088 | 1 | 7 | 240 | 8 | 0.137018 | 1 |
| 11 | 160 | 16 | 0 | 0 | 7 | 240 | 8 | 0.14463 | 0.992461 |
| 11 | 160 | 16 | 0.011418 | 0.012084 | 7 | 240 | 8 | 0.152242 | 0.992461 |
